# Paediatric Autism Communication Therapy-Generalised (PACT-G) against treatment as usual for reducing symptom severity in young children with autism spectrum disorder: study protocol for a randomised controlled trial

**DOI:** 10.1186/s13063-018-2881-3

**Published:** 2018-09-21

**Authors:** Jonathan Green, Catherine Aldred, Tony Charman, Ann Le Couteur, Richard A. Emsley, Victoria Grahame, Patricia Howlin, Neil Humphrey, Kathy Leadbitter, Helen McConachie, Jeremy R. Parr, Andrew Pickles, Vicky Slonims, Carol Taylor, PACT-G Group, Matea Balabanovska, Hilary Beach, Claire Bennett, Sophie Carruthers, Imogen Crook, Hannah Danvers, Kate Dartnall, Ceri Ellis, Hannah Foote, Jessica Graham, Kirsty James, Sarah Jamieson, Anna Knight, Jo Lowe, Ruth Madeley, Olivia Mitchell, Francisca Monteiro, Heather L. Moore, Helen Morley, Jessica Rose, Lauren Taylor, Su Vosper

**Affiliations:** 1 Division of Neuroscience and Experimental Psychology, University of Manchester, PACT-G Trial Office, Room 3.312, Jean McFarlane Building, Oxford Road, Manchester, M13 9PL UK; 2 Children’s and Young People’s Disability Partnership, Kingsgate House, Wellington Road North, Stockport, SK4 1LW UK; 3 Department of Psychology, Institute of Psychiatry, Psychology and Neuroscience, Box PO77, Henry Wellcome Building, De Crespigny Park, Denmark Hill, London, SE5 8AF UK; 4 Institute of Health and Society, Sir James Spence Institute, Royal Victoria Infirmary, Level 3, Queen Victoria Road, Newcastle upon Tyne, NE1 4LP UK; 5 Department of Biostatistics and Health Informatics, Institute of Psychiatry, Psychology and Neuroscience, Room S 2.03, De Crespigny Park, London, SE5 8AF UK; 6 Northumberland, Tyne and Wear NHS Foundation Trust, Walkergate Park, Benfield Rd, Newcastle upon Tyne, NE6 4QD UK; 7 Manchester Institute of Education, University of Manchester, Ellen Wilkinson Building, Oxford Road, Manchester, M13 9PL UK; 8 Royal Manchester Children’s Hospital, Manchester University NHS Foundation Trust, Oxford Road, Manchester, M13 9WL UK; 9 Institute of Neuroscience, Newcastle University, Framlington Place, Newcastle upon Tyne, NE1 9DU UK; 10 Institute of Psychiatry, Psychology and Neuroscience, Room S 2.10, De Crespigny Park, London, SE5 8AF UK; 11 Guy’s and St Thomas’ NHS Foundation Trust (Evelina Children’s Hospital), Newcomen Centre at St Thomas’ 2nd Floor, Becket House, 1 Lambeth Palace Road, London, SE1 7EU UK

**Keywords:** Autism spectrum disorder, Randomised trial, Social communication intervention, School-based intervention

## Abstract

**Background:**

Prior evidence shows that behaviours closely related to the intervention delivered for autism are amenable to change, but it is more difficult to generalise treatment effects beyond the intervention context. We test an early autism intervention designed to promote generalisation of therapy-acquired skills into home and school contexts to improve adaptive function and reduce symptoms. A detailed mechanism study will address the process of such generalisation. Objective 1 will be to test if the PACT-G intervention improves autism symptom outcome in the home and school context of the intervention as well as in the primary outcome research setting. Objective 2 will use the mechanism analysis to test for evidence of acquired skills from intervention generalizing across contexts and producing additive effects on primary outcome.

**Methods/design:**

This is a three-site, two-parallel-group, randomised controlled trial of the experimental treatment plus treatment as usual (TAU) versus TAU alone. Children aged 2–11 years (*n* = 244 (122 intervention/122 TAU; ~ 82/site) meeting criteria for core autism will be eligible. The experimental intervention builds on a clinic-based Pre-school Autism Communication Treatment model (PACT), delivered with the primary caregiver, combined with additional theory- and evidence-based strategies designed to enhance the generalisation of effects into naturalistic home and education contexts. The control intervention will be TAU.

Primary outcome: autism symptom outcome, researcher-assessed using a standardised protocol. Secondary outcomes: autism symptoms, child interaction with parent or teacher, language and reported functional outcomes in home and school settings. Outcomes measured at baseline and 12-month endpoint in all settings with interim interaction measurements (7 months) to test treatment effect mechanisms.

Primary analysis will estimate between-group difference in primary outcome using analysis of covariance with test of homogeneity of effect across age group. Mechanism analysis will use regression models to test for mediation on primary outcome by parent-child and teaching staff-child social interaction.

**Discussion:**

This is an efficacy and mechanism trial of generalising evidence-based autism treatment into home and school settings. It will provide data on whether extending treatment across naturalistic contexts enhances overall effect and data on the mechanism in autism development of the generalisation of acquired developmental skills across contexts.

**Trial registration:**

ISRCTN, ID: 25378536. Prospectively registered on 9 March 2016:

**Electronic supplementary material:**

The online version of this article (10.1186/s13063-018-2881-3) contains supplementary material, which is available to authorized users.

## Background

### Introduction

Intervention research in autism spectrum disorder (ASD) (hereafter ‘autism’) has recently accelerated, with studies across a range of interventions considered in recent NICE guidance [1], Cochrane [2] and other reviews [3–5]. The pattern of findings across a number of interventions is for reproducible moderate to good effects on targeted proximal outcomes such as improvement in interaction and communication in the treatment context [6, 7] but there is much less evidence for generalisation of treatment effect to broader symptom change and functional outcome [3]. This problem of generalising from ‘proximal’ intervention effects to wider symptom and functional change is a key current challenge for autism treatment research [4, 8]. The capacity to generalise acquired skills flexibly across contexts is a central feature of successful developmental learning, and is a major problem for individuals with autism [9]. *Parent-mediated and education staff-mediated learning*, providing the same dyadic cues for the child across different contexts, is one plausible approach to helping overcome these generalisation difficulties in autism [10]. *Naturalistic learning,* in which the learning takes place within the functional context in which the skills are actually needed, provides another potential approach. Working with children in their natural environments is now often highlighted as best practice for early intervention [11].

Early social communication intervention, delivered through parents, therapists or teachers, is the only autism treatment currently recommended for consideration by NICE [1]. The Pre-school Autism Communication Trial (PACT) tested a clinic-delivered, parent-mediated social communication intervention against regular care in one of the largest randomised controlled trials (RCTs) yet in the field [12]. The therapy showed a substantial impact on the targeted immediate outcome of parental communicative synchrony with the child (ES 1.22 (95% CI 0.85, 1.59) and also on the child’s communication initiations with the parent (ES 0.41 (0.08, 0.74). Autism symptoms spanning social communication and restricted and repetitive behaviours (measured with a different interaction partner in a different structured context) were significantly reduced at treatment endpoint (ES 0·64, 95% CI 0·07, 1·20) and at 6-year follow-up (ES 0·70, 95% CI − 0·05, 1·47), resulting in a significant overall effect over the treatment and follow-up period (ES 0·55, 95% CI 0·14, 0·91). Non-blind parent-rated autism symptoms on the Social Communication Questionnaire (ES 0·40, 95% CI 0·05, 0·77) and repetitive behaviours on the Repetitive Behaviour Questionnaire (ES 0·87, 95% CI 0·47, 1·35) also showed comparable improvement at follow-up [12].

The current Paediatric Autism Communication Therapy-Generalised (PACT-G) trial now builds on this clinic-based work by using additional strategies that are designed to improve generalisation of the effects demonstrated into wider symptom change and functional impact in other environments. It does this by incorporating parent- and education staff-mediated intervention strategies within the naturalistic learning contexts of home and school/nursery. A further development is to extend the application of the intervention into the primary school years. Autism intervention studies to date have been largely limited to episodic interventions, usually in pre-school. However, communication skills continue to emerge and develop beyond the pre-school years [13] and social communication skills in the early-school-age period are strong predictors for later development [14]. The persisting and significant impairments in social interaction and communication among children with autism argue strongly for a developmentally sustained approach to intervention into middle childhood in affected children. Additionally, a mechanism study within the PACT-G trial will build on the understanding gained from the design and mediation analysis in the original PACT trial [15] by assessing the mediators and outcomes in the different generalisation contexts, and thus provides a unique and innovative opportunity to further understand the processes and facilitation of symptom change in autism.

## Methods/design

### Aims

The PACT-G study has two aims. Firstly, it will test whether the extended PACT-G social communication intervention protocol, using targeted enhancement strategies within home and education settings, improves transmission of treatment effect to:

(1) Researcher-assessed autism symptom outcome; (2) Autism symptoms and functional adaptation in home and education settings. This objective will be tested using blinded measures maximising the ability to detect meaningful change (see ‘Measures’ below) and evaluated by analysis at trial endpoint.

The second aim is a mechanism analysis that will use the experimental trial to illuminate core processes of generalisation of specific acquired competencies in autism across context: (1) We will build on the mediation analysis from our previous PACT Trial (see above) to test mediation of the generalised treatment effect in home and school, (2) We will test how effects in naturalistic contexts may combine to enhance transmission of effect to research-assessed symptoms in a standardised test setting. In doing this, we will use the pre-specified measures of mediation which were successful in our previous MRC PACT trial.

### Design

A three-site, two-group, randomised controlled trial of the experimental treatment plus TAU compared to TAU alone. Children aged 2–11 years with core autism will be recruited to the trial in the local areas following referral via clinical specialists, education professionals and consented databases. After consent families will be randomised in three sites around the UK to receive either the PACT-G social communication intervention in addition to TAU or TAU alone**.** Assessments are administered on entry (baseline) to the trial, at the 7-month midpoint and at the 12-month endpoint. There will be an embedded mechanism study to test mediation hypotheses and illuminate the basic science underpinning the understanding of generalisation impairments in autism.

### Settings

NHS clinics, homes, local schools with specialist autism units, and specialist autism school settings; in Greater Manchester, London and North-East England.

### Study population

#### Inclusion criteria

- Age 2–11 years
- Diagnosis of autism spectrum disorder (ASD)
- Meeting criteria for autism on the Autism Diagnostic Observation Schedule-2nd Edition (ADOS-2) [16] and scoring ≥ 15 (school-aged) and ≥ 12 (pre-school) on the Social Communication Questionnaire (SCQ) [17]
- Children who are aged 5 years and over are between P3 and P8 for the English curriculum [18] (as reported by relevant professionals; the P levels were designed to be used for pupils with learning disability. P3 communication skills indicate that a child is beginning to use ‘intentional communication’. P8 represents up to, but not beyond, a language age equivalent of 4 years in a typically developing child)
- Parents with sufficient English to potentially participate in the intervention and who speak English to their child at least some of the time

#### Exclusion criteria

- Sibling with autism already in the trial
- Participation in the PACT-G pilot phase
- Children aged ≤ 12 months non-verbal age-equivalent level
- Epilepsy not controlled by medication
- Severe hearing or visual impairment in parent or child
- Current severe learning disability in the parent, or current severe parental psychiatric disorder
- Current safeguarding concerns or other family situation that would affect child/family participation in the trial
- No agreement to participate from child’s education setting
- Children with an identified genetic disorder that would impact on ability to participate or affect validity of data; eligibility to be determined by the principal investigators (PIs) on a case-by-case basis

### Treatment principles

PACT-G is an enhancement of the original clinic-based PACT therapy. This is a ‘carer-mediated’ therapy in which caregivers are coached, using video-feedback, to interact with the child using evidence-based strategies that facilitate social communication development in the child. Optimal interaction with a sensitive and responsive communication partner (such as the parent/caregiver) increases communication and social interaction skills in the child. In the original PACT trial this approach was found to be very effective in increasing the quality of parental communicative responses to the child, which in turn led to increased child-initiated social communications with the parent.

PACT-G retains these effective elements but adds new features to aid the generalisation of the child’s newly acquired skills into other settings, recognising that such generalisation is a particular problem in autism. PACT-G encourages generalisation of skills by extending the therapy into the home and school settings, by integrating the parental techniques into daily routines and play, and by widening the range of adults involved in training to include education staff in addition to parents/carers. The therapy generally begins with the parent at home then extends into the educational setting but flexibility in timing is built in to fit with school terms, with an overlap to allow for essential supported joint collaboration with parent and education staff.

PACT-G has also been modified to incorporate recent advances in research, focussing on specific strategies to enhance the child’s response to adult-directed shared attention and to develop object interest and play. These are important precursors to the early stages of language development [19, 20] and have been shown to moderate treatment response in recent social communication early autism trials [21]. Further modifications allow more individual differentiation so that intervention begins at a point appropriate to the child’s initial level of object interest and social engagement.

PACT-G, in common with the original PACT therapy, takes a staged approach, which is based on theoretically informed child developmental progression and strategies for establishing essential foundation skills such as shared attention. Parents and education staff are helped to recognise and facilitate child motivated play and increase their synchrony and sensitive responding (stages 1–2) with verbal comments on child action and play. Middle stages (stages 3–4) of PACT-G develop language comprehension and expression through commenting on the child’s activity, language ‘mapping’ and modelling, and encourage child communication initiations through the use of anticipation and other eliciting techniques. For children who make the most progress, later stages (stages 5–6) encourage language expansion and conversation. PACT-G is appropriate for pre-school and also primary-school-age children who have lower functioning autism. Some children are likely to be at the earliest stages of communication development, making the early developmental PACT-G stages focussing on shared attention, adapted parent/ education staff responding and eliciting child communication initiation appropriate. Other children may be verbally fluent making appropriate the later PACT-G stages, which focus on language understanding, expression, language expansions and conversation.

**Fig. 1:**
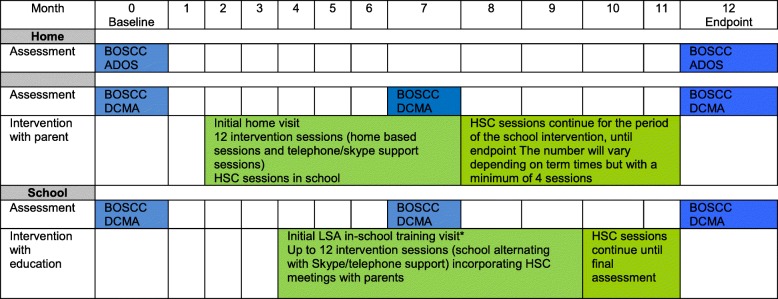
Intervention and assessment timeline. Legend: *Start of education element accommodates school terms. Key: *BOSCC* Brief Observation of Social Communication Change, *ADOS* Autism Diagnostic Observation Schedule-2; *HSC* Home-School Conversation (see text)

The sequence of delivery of the PACT-G intervention is set out visually in Fig. 1.

#### Parent sessions

Based on what was found to be most effective in the original PACT trial, parents will receive 12 intervention sessions. Prior to starting the intervention, a home visit is conducted to introduce the intervention to the parents, explore the family context and set expectations. The therapy sessions are delivered in the home. Subsequent sessions alternate between home-based sessions and Skype/telephone-delivered consultation. Delivery is flexible in accordance with the needs of the family. This approach will assist generalisation of new skills development in the home setting. Clinical and research experience indicates that these session formats are popular with parents [22]. Each parent session begins with a discussion of progress made since the last session. The parent and child are then filmed whilst playing for 10 min. Immediately after this the therapist and parent watch the film, or during Skype sessions the therapist and parent watch a 1–2 min parent-made video of a home-based practice session routine. The therapist facilitates the parent to identify actions that lead to child communication and to adopt PACT-G strategies in their interaction with the child. Parents are assisted to set goals for themselves, based on the interaction strategies discussed. The parent and therapist discuss the opportunities to practice these strategies each day outside of therapy sessions and parents are asked to make time to practice them daily for half an hour.

#### Education-setting sessions

In most cases, therapy in the educational setting begins after the parent has commenced therapy at home. The start times and duration of education-based therapy are flexible to fit around the school-term schedule. In the education setting PACT-G sessions will be delivered to trained learning support assistants (LSA), who are staff with a specific remit to assist children with special educational needs to access the curriculum- and broader school-based activities, e.g. mealtimes, etc. LSAs and other education staff receive an initial training session to introduce them to PACT-G. The education-based intervention then consists of therapist-LSA sessions that mirror the therapist-parent sessions in the home. Videos are made of the LSA and the child and are used to coach the LSA in the use of PACT-G strategies in a similar procedure to the parent. The LSA then implements these with the child daily in class time. There are a maximum of 12 therapist-LSA sessions over 6 months, alternating in-school visits and Skype/telephone consultation. PACT-G strategies are expected to be integrated in a complementary way with other communication strategies that may already be in use in the school.

#### Collaboration between parent and educational staff

Importantly, the separate therapeutic work with parents and LSAs described above will be supplemented with a schedule of joint parent-LSA meetings to support the work and ensure consistent use of strategies across home and education settings. This is hypothesised as being key to successful generalisation. The meetings will use the manualised technique of ‘Home-School Conversation’ (HSC). Meetings are structured around ‘explore’, ‘focus’, ‘plan’ and ‘review’ stages, which allow the LSA and parent to share experiences and maximize intervention consistency. HSC is validated and shown to be highly effective in motivating parents and school staff [23, 24].

### Training and fidelity of treatment

Training in the PACT-G will be conducted centrally by the lead speech and language therapists, who will undertake overall co-ordination of the therapy in the trial and will organise quarterly across-site therapist meetings. Therapists will be regularly supervised by the lead speech and language therapists in each site. All therapy sessions will be videotaped and 5% of randomly selected tapes will be independently rated using the PACT Fidelity Rating Scale at regular intervals across the trial period. Therapists in the trial will not be treating any TAU patients. Therapists and research staff will be trained in practices that minimise non-compliance and drop-out. Therapy compliance and receipt of other interventions outside of the protocol will be monitored.

### Treatment as usual and avoidance of contamination

The control intervention will be treatment as usual (TAU). We have detailed information on TAU in the pre-school population from the group’s previous work on the MRC PACT trial and in older children from the PACT 7–11 follow-up study [25]. Data on services received will be collected.

There will be separate clinical and research leads at each site and separate training and supervision structures. Researchers will be housed separately from staff involved in delivery of the PACT-G intervention. Mid- and endpoint research interviews and assessments will be conducted so as to avoid inadvertent divulging of information that could infer treatment status. The assessment suite and materials used will be quite different in type and location to that used for the therapy intervention in home or education, avoiding any familiarity effect for children in the treatment arm.

### Measures (see Fig. 2)

#### Primary outcome

##### Autism Diagnostic Observation Schedule (ADOS-2) [16, 26]

The standard autism diagnostic symptom measure with good external validity to long-term outcomes in autism development. Measured within researcher-child interaction using a standardised set of social presses, video-recorded for later coding. The scoring metrics of ADOS have been modified in line with the 2013 DSM-5 [27], with social communication and repetitive behaviour symptom domains combined into a unitary total symptom score (Social Affect + Restricted and Repetitive Behaviour Overall Total raw score). Recent studies [12, 28] have demonstrated the ability of the ADOS to measure treatment effects; for instance in the PACT trial [12] finding effects sustained 6 years after treatment end.

**Fig. 2:**
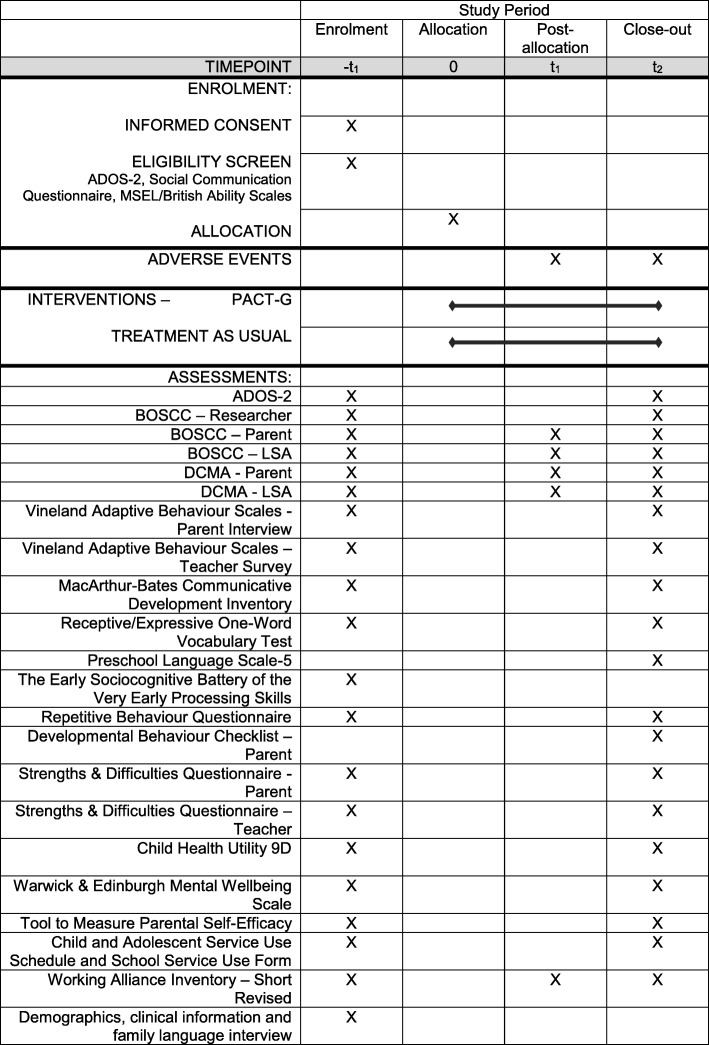
Schedule of assessments. Key: *ADOS* Autism Diagnostic Observation Schedule-2, *MSEL* Mullen Scales of Early Learning, *BOSCC* Brief Observation of Social Communication Change, *DCMA* Dyadic Communication Measure for Autism

### Other measures

#### Diagnostic inclusion

##### Mullen Scales of Early Learning [29] or British Ability Scales [30]

Depending on child age and ability level. Standard measures of non-verbal early development which enable a developmental level of non-verbal abilities to be ascertained for inclusion criteria and allow characterisation of the cohort in relation to other autism treatment trials.

##### Social Communication Questionnaire (SCQ) Lifetime Version [17]

A brief (40-item), parent-report screening measure that identifies characteristics associated with ASD. Items cover three subdomains: Reciprocal Social Interaction, Communication, and Restricted, Repetitive and Stereotyped Patterns of Behaviour. The ‘lifetime’ version of the SCQ refers to the entire developmental history of the child.

#### Mechanism testing

##### Brief Observation of Social Communication Change (BOSCC) with researcher [31–33]

A researcher-coding of autism symptoms from videotaped child-adult interaction. It addresses the same autism symptom construct as ADOS but is designed to better detect clinically meaningful symptom change in treatment studies, with codes combining symptom frequency, severity and atypicality on a 16-item, 0–5 scale (overall range 0–80). The BOSCC is designed to be a standard treatment outcome measure for the autism field and is currently used in large funded trials in the US and the EU. It shows high inter-rater agreement [31] and increased sensitivity to treatment change compared to ADOS (BOSCC *d* = 0.64 compared to parallel ADOS *d* = 0.42 in a recent 12-month observational intervention study) [34].

##### Brief Observation of Social Communication Change (BOSCC) with parent and LSA [31–33]

Coded from video of child-parent play-session in home (baseline, 7-month midpoint, 12-month endpoint) and child-learning support assistant in school (baseline, 7-month interim, 12-month endpoint); measure of intervention effect in naturalistic settings in which intervention took place.

##### Dyadic Communication Measure for Autism (DCMA) with parent and with LSA [12]

Coded from video of the child-parent play-session at home (baseline, 7 months midpoint, 12-month endpoint) and child-learning support assistant play-session in school (baseline, 7 months interim, 12-month endpoint). This measure includes independent codes of parental communication (synchrony) and child communication (initiations). This measure proved sensitive in the original PACT mediation analysis and will be used in PACT-G to test treatment effect and mediation in home and education settings.

#### Secondary outcome

##### Vineland Adaptive Behaviour Scales

Parent and teacher versions (P/T-VABS) [35]. The VABS includes domains of communication, daily living skills and socialisation, and has been used in numerous autism studies. It will be a measure of functional gains by the child in the home and education settings.

##### MacArthur-Bates Communicative Development Inventories (Word and Gestures; Sentences and Grammar) [36]; and Receptive and Expressive One-word Picture Vocabulary Test [37]; and Pre-school Language Scale-5 [38]

The overall language level measured by these standardised assessments supplements that of the measures of autism-specific communication included in the BOSCC and ADOS.

##### The Early Sociocognitive Battery of the Very Early Processing Skills [39, 40]

This assesses children’s sociocognitive skills (social responsiveness, joint attention and symbolic comprehension).

##### Repetitive Behaviours Questionnaire [41]

A 26-point parent questionnaire for assessing repetitive behaviours in children with ASD.

##### The Developmental Behaviour Checklist-Parent (2nd Edition; DBC-P) [42] Disruptive/Anti-social and Anxiety Subscales

A 96-item instrument used for the assessment of behavioural and emotional problems in young people aged 4–18 years with developmental and intellectual disabilities. It is completed by a parent or carer. In PACT-G we will use two subscales: the Disruptive/Anti-social and the Anxiety Subscale. This constitutes 36 items.

##### Strengths and Difficulties Questionnaire (SDQ) – Parent and Teacher versions

A 25-item brief measure of psychological wellbeing in 2–17 year olds (Goodman, 1997) [43]. In PACT-G, it will be completed by both parents and teachers.

##### Child Health Utility 9D [44] (CHU9D)

A paediatric measure of health-related quality of life. It consists of nine items, each responded to with one of five levels (ranging from no problems to severe problems). The CHU9D is designed to be completed by children aged 7–17 years. Proxy completion is also possible for younger/developmentally disabled children. In PACT-G parents will be asked to complete this questionnaire on behalf of their child.

##### Warwick and Edinburgh Mental-Wellbeing Scale [45]

Parent-rated wellbeing questionnaire recommended by UK Department of Health as the preferred measure of mental wellbeing important to incorporate in studies of this kind.

##### Tool to Measure Parental Self-efficacy [46]

A 48-item, self-report measure of parenting competence. It is a measure of possible change in parent’s confidence in their ability to make a difference to their child’s development. Completed at baseline and endpoint assessments.

##### Child and Adolescent Service Use Schedule (CA-SUS) [25] and School Service Use Schedule

Developed to record therapies and service use accessed throughout participation in the study. Forms were adapted to young populations with autism in our PACT and PACT 7–11 studies [6].

##### Working Alliance Inventory – Short Revised (WAI-SR) [47]

A measure of engagement with therapy for parents and learning support assistants in intervention group only. For parents and LSAs, there is a simple rewording of the client and therapist versions of the WAI-SR, which has been validated and is now frequently used. This will be completed at 2 and 5 months into the intervention.

##### Demographic, clinical and family language information

We will collect relevant demographic and clinical information and details of home language(s) spoken with the child.

### Procedures

#### Data collection

Research staff will confirm eligibility and obtain consent. Baseline assessment will be undertaken prior to treatment assignment. Randomisation will be done through the King’s Clinical Trials Unit web-based randomisation service. Allocation will be by stratified block randomisation, controlling for treatment centre, age strata (under fifth birthday, fifth birthday and older) and gender. Each case will be assigned a participant ID number and treatment allocation emailed separately to the treatment centre therapists.

The primary outcome and putative mechanisms are coded from videotape, by researchers at the other sites, trained to high levels of reliability and blinded to intervention allocation. A randomly selected proportion of assessments will be double rated for reliability. All other researcher assessments are also blinded; parent and teacher questionnaires/interview measures non-blinded. Participant families cannot be blinded to allocation.

All therapy sessions are videotaped. Variability due to therapist effects will be minimised by frequent clinical supervision and checks on continuing therapist fidelity against the treatment manual; randomly selected sessions for each therapist will be formally coded for fidelity over the course of the study by independent clinicians using the model successfully used in PACT.

Adverse events are enquired for by researchers at each contact with the family. Adverse events are also collected in parallel by trial therapists, as and when a situation becomes known to them, and documented separately. As well as recording adverse events in a pre-defined standard format, we include adverse events relating to child health, wellbeing and behaviour, significant issues at school, and family events such as separation or significant parental ill-health.

Extensive information-sharing and engagement activities with clinical teams and local mainstream and specialist schools will be undertaken to promote clinical referrals and engagement with both home and education aspects of the intervention. Regular trial newsletters to participating families, schools and nurseries and clinical teams, along with voucher payments to schools and nurseries will act to maintain involvement and adherence. Families receive an individualised feedback report on the assessments conducted with their child, copied to school and clinical teams if desired. A local referring clinician for each participant will be informed of study progress and findings, with procedures for clinical support and aftercare beyond the study should this be necessary.

#### Data management

All data in the trial will be anonymised. A central master file will be held by the trial manager at The University of Manchester. This will contain the key linking anonymised trial name to personal details. The main trial data will be entered into the web-based data entry service of King’s College Clinical Trials Unit, which has a full audit trail. Appropriate quality control will be carried out during the trial and before the database lock.

Primary analysis of the data will take place by the trial statisticians and chief investigator. Other members of the team will also have access to data within a publication protocol agreement, and will be able to undertake analysis as appropriate and necessary. This could include analysis of baseline data prior to primary endpoint analysis, and of outcome data but only after the primary analyses are completed. Any arrangements for other researchers in the general field to have access to the primary data will be negotiated separately and COREC informed.

## Statistical analysis

### Sample size calculations

The PACT trial showed an effect of ES 1.22 (0.85, 1.59) on parental synchrony (DCMA), which mediated 70% of the ES 0.41 (0.08, 0.74) on child communication, which in turn mediated 72% of the ES 0.24 (0.59, 0.11) on symptom outcome (ADOS). The intervention strategies in the PACT-G trial are specifically targeted to enhance generalisation of the child communication to increase effects on primary outcome in home, education and research settings. Therefore, we expect the ES for the symptom outcome to be substantially above 0.24 and clinically meaningful (see above). Power was calculated using the sampsi command in Stata for an analysis using analysis of covariance (ANCOVA) with alpha = .05, with pre and post measures correlated at .67 (based on PACT trial). With 110 cases followed up in each group (70/70 pre-school and 40/40 school-age) 80% power is retained for ES = 0.28 and 90% power for ES = 0.33. Allowing for 10% attrition (compared to 4% in PACT) we propose to recruit 244 families (~ 82/site − 52 pre + 30 school-age).

### Analysis plan

A statistical analysis plan will be written by the trial statisticians (AP and RE) and agreed by the trial principal investigators before submitting for approval by the Trial Steering Committee and Data Monitoring Committee before any analysis is undertaken. All statistical analyses will be carried out using the latest version of Stata [48] or MPlus [49].

In accordance with the Consolidated Standards of Reporting Trials (CONSORT) Statement for non-pharmacological interventions, we will report all participant flow. Descriptive statistics will summarise recruitment, drop-out and completeness of interventions. Analysis will be undertaken after all 12-month outcome measures are completed. The main efficacy analysis will follow intention-to-treat principles. For the primary outcome, baseline measurement will be complete, since required for randomisation. The proposed ANCOVA will provide estimates under an assumption of a missing-at-random (MAR) data mechanism. For secondary outcome measures the ANCOVA will be estimated using simultaneous equations for both baseline and follow-up measurements, including all participants with any pre- or post-randomisation measure under the MAR assumption. Where outcomes are unavailable on more than 10% of participants, a sensitivity analysis will be undertaken in which outcome scores will be multiply imputed using relevant available auxiliary baseline and follow-up measures and assuming the absence of a treatment effect. There will be no planned interim analysis for efficacy or futility. Summary statistics of baseline characteristics will be presented by randomised group without formal statistical tests.

#### Phase 1 – Efficacy analysis

We will test the primary hypothesis for between-group difference in the outcome ADOS total score using linear regression, stratified by ADOS module, covarying by baseline ADOS total score and dummy variables for site, gender and age group. Standard residual diagnostics will be applied and skew minimising transformations adopted where required. An overall effect size will be calculated pooling stratum specific estimates for strata defined by the ADOS module, weighted by their precision, using a 95% confidence interval estimated from 5000 bootstrap replicates.

The secondary outcomes will be analysed in a similar way but without stratification by ADOS module. A forest plot of effect sizes for primary and secondary outcomes will be presented. A test of homogeneity of effect size for the ADOS and BOSCC will be reported.

The primary paper will report a test of homogeneity of effect for the primary outcome in pre-school and school-age children. To be consistent with the treatment main effects analysis, the test of difference in treatment effect by age group will be based on the bootstrap *p* value over 5000 replicates of the pooled within-ADOS stratum estimate of the treatment difference. A secondary paper (see phase 3) will report an optimal moderation index [50] including bias correction from over-fitting to a finite sample.

#### Phase 2 – Mechanisms’ evaluation

**Fig. 3:**
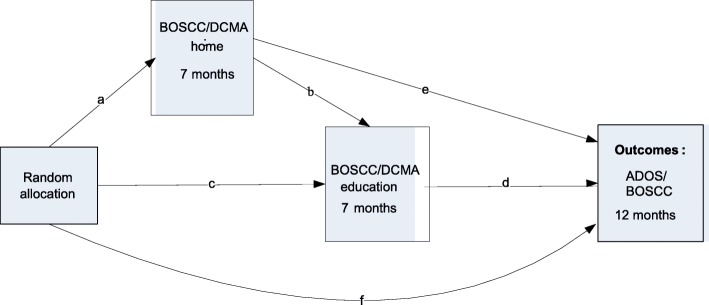
Key mediation pathways to be tested in the Paediatric Autism Communication Therapy-Generalised (PACT-G) Trial mechanism study

Mediation analysis [15] gave detailed insight into an attenuated generalization in the original PACT trial across change in person, task and context (as above and Fig. 1). In the PACT-G trial we enhance generalisation *into home* by keeping parental dyadic cues constant, but increasing functionally relevant interaction contexts; and *into education* by enhancing relevant communication with education staff (LSA). The mechanism study will investigate the mediation process in this model and, through that, illuminate key basic knowledge about generalisation of acquired skills in autism. Some of the pathways of interest are illustrated in Fig. 3. If the efficacy analysis shows significant between-group differences in the mediators (DCMA and/or BOSCC at home (path a) and in education contexts (path c)), then we will use parametric regression models to:

1. Test for mediation of the intervention on primary symptom outcome (ADOS) through DCMA and/or BOSCC at home (paths a,e,f)
2. Test for mediation of the intervention on primary symptom outcome through DCMA and/or BOSCC in the education setting (paths c,d,f)
3. Test for mediation of intervention on DCMA and/or BOSCC in education setting through DCMA and/or BOSCC at home (paths a,b,c)
4. Use structural equation modeling to examine multiple pathways through DCMA and/or BOSCC at home and in the education setting to primary symptom outcome (paths a–f)

We will also repeat these four steps using researcher BOSCC as the outcome variable in place of the ADOS. Since all the measures are continuous, the indirect effects are calculated by multiplying relevant pathways and bootstrapping is used to produce valid standard errors for the indirect effects. All analyses will adjust for baseline measures of the mediators (BOSCC/DCMA), outcome (ADOS) and putative measured confounders. Mediation analyses are potentially biased by measurement error in mediators and hidden confounding between mediators and outcomes; we will build on our previous methodological and applied work in this context to include repeated measurement of mediators and outcomes to account for classical measurement error [15] and baseline confounding [51]. We will investigate the sensitivity of the estimates to these problems and that of unmeasured confounding using instrumental variable (IV) methods [52] with baseline covariate by randomization interactions as potential instruments [52].

Treatment compliance in the education setting is likely to be more variable than the high levels achieved with parents. We will estimate a complier average causal effect (CACE) estimate using instrumental variable methods, considering the extent of education-setting opt-in as a measure of compliance and randomisation as the instrumental variable.

#### Phase 3 – Moderation and subgroups

We will test whether the mediation analysis is consistent across the two age groups by testing for moderation of paths a–f by age-group stratifier (including interaction terms or performing a multiple group analysis in the structural equation model). We will test ‘*moderated mediation*’ on our pathway from intervention to interaction with an unfamiliar assessor, extending our understanding of generalisation processes in autism. The heterogeneity of autism is well-recognised and as such offers numerous potential moderators of treatment effects (e.g. language level, restricted and repetitive behaviour, functional impairment). We will examine an extended list of moderators using bias correction/cross-validation methods to identify robust evidence for moderation and for a moderation index, both on the overall effect and also along the steps of the mediation pathway.

## Discussion

This trial addresses a number of contemporary challenges in autism intervention research. Firstly, it addresses the challenge of implementation of a clinic-based treatment into the naturalistic community contexts of home and education. The logic for undertaking this generalised implementation is to address a second key difficulty within autism development; that is the difficulty that children with autism have in generalising acquired skills across context and person. This barrier to generalisation in autism has been a significant issue limiting the everyday and functional effectiveness of interventions for child adaptation and development. Thirdly, the study extends the age of primary intervention into the early school years, whereas most early psychosocial interventions to date have been implemented in the pre-school period.

The study will take a rigorous approach to outcome assessment with objectively measured autism symptoms (i.e. the defining characteristics of the disorder) as the primary outcome. It will use new techniques in objective measurement of autism symptomatology in naturalistic contexts to test the differential effect of intervention in home and school, and any additive effects that may occur. Secondary outcomes will test a range of parent- and teacher- rated outcomes relating to development, behaviour, adaptation, parental wellbeing and self-efficacy and family quality of life. These are designed to capture the effect of intervention in the widest possible way within the child’s development and social and family context.

The trial and the mechanism analysis will allow the testing of how effects in different naturalistic contexts relate together and contribute to overall developmental outcomes. This will result in novel and important information relating to the issue of the generalisation of acquired skills in autism and the impact of this on development and susceptibility to treatment.

Implementation of a video-aided intervention of this kind in the home and school context will be challenging since many aspects of the environment in both these contexts may interfere with the focus necessary for therapeutic work. These challenges have to be balanced against the potential advantages of naturalistic context intervention. We have built in operational features to try and mitigate these challenges and have also included within the protocol a regular structured collaborative conversation between parents and teachers with the aim of enhancing the integration of the parallel home and education treatment programmes. In doing this work the trial will address core current NHS priorities in child development and mental health, for instance the emphasis on evaluation of school-based intervention in recent health policy. The three-site design of the study, in major urban/semi-urban geographically disparate centres will, from our previous work, provide a useful range of geographic coverage and social demographic representation for the study design. The trial’s focus on autism symptoms as the primary outcome is consistent with our previous work, addressing the core defining characteristics of the disorder. We will utilise new leading measurement techniques in autism symptom outcome estimation. The data-rich, repeated-measures design will give enhanced power to account for the inevitable measurement error in behavioural studies of this kind and allow sophisticated mediation/mechanism modelling of the findings. The outcomes of the trial will add important information to our knowledge about effective interventions for autism development (Additional file 1).

## Trial status

Protocol: Version 6. 12 March 2018.

Recruitment began on 18 January 2017 and completed on 30th April 2018.

## Monitoring

### Sponsor and monitor

Manchester University NHS Foundation Trust, Dr. Lynne Webster, Central Research Office, Nowgen Building, Grafton Street M13 9WL. Telephone: 01612764125.

### Trial Steering Committee

Composition: Professor Stuart Logan, University of Bristol (Chair); Professor Anne O’Hare, University of Edinburgh; Professor Liz Pellicano, Macquarie University, Sydney (resigned); Dr Emily Jones, Birkbeck College London; Ms Louisa Harrison (parent representative); Ms Kellie Bell (parent representative); Professor Jonathan Green, University of Manchester (CI). The TSC is independent of sponsor and funder and declares no competing interests.

### Data Monitoring and Ethics Committee

Composition: Professor Paul Ramchandani, University of Cambridge (chair); Professor Amanda Farrin, University of Leeds; Professor Jacqueline Barnes, Birkbeck College, London; Professor Andrew Pickles (PI statistician), King’s College London. The DMEC is independent of sponsor and funder and declares no competing interests. Further details of the DMEC charter are available from Professor Andrew Pickles, King’s College London. There is no planned formal interim analysis.

### Project Management Group

A Project Management Group will be chaired by CI Professor Green and consist of the principal investigators and senior researchers on the trial, the trial manager and other invited members as necessary. It will meet at least quarterly, with additional tele or video-conferencing as necessary.

### Harms

We will actively collect information at each assessment point of the trial about adverse events. In addition to recording adverse events in the standard way, we will include events particularly relevant to this trial, such as significant changes in family or school situation. There are standard operating procedures for reporting serious adverse events to the Project Management Group, TSC, DMEC, sponsor and funder, for consideration of appropriate action.

### Auditing

Trial conduct is monitored by regular auditing visits from the sponsor, annual reports to the NHS Research Ethics Committee (REC), bi-annual reports to the funder and regular Trial Steering Committee meetings. Protocol amendments will be formally recorded and communicated to the Project Management Group, REC, funder (NIHR EME), DMEC, TSC and reported in the trial registration site.

## Dissemination

The results of the research will be targeted for publication in peer-reviewed journals of general and special interest. There will also be a general dissemination programme for families including participants co-ordinated through our collaborators in the National Autistic Society. Individual feedback for participants will be through the regular trial newsletter. Authorship on dissemination papers will follow ICMJE guidelines and journal requirements. There will be no use of professional writers.

## Additional file

Additional file 1: Standard Protocol Items: Recommendations for Interventional Trials (SPIRIT) 2013 Checklist: recommended items to address in a clinical trial protocol and related documents*. (PDF 107 kb)

## Abbreviations

ADOS: Autism Diagnostic Observation Schedule-2
ASD: Autism spectrum disorder
BOSCC: Brief Observation of Social Communication Change
COREC: Central Office of Research Ethics Committees
DCMA: Dyadic Communication Measure for Autism
HSC: Home-School Conversation
LSA: Learning support assistant
MRC: Medical Research Council of United Kingdom
MSEL: Mullen Scales of Early Learning
NHS: United Kingdom National Health Service
NICE: United Kingdom National Institute for Health and Care Excellence
PACT: Pre-school Autism Communication Therapy
PACT-G: Paediatric Autism Communication Therapy-Generalised
SCQ: Social Communication Questionnaire
TAU: Treatment as usual
